# Programmed Cell Death-1 Receptor (PD-1)-Mediated Regulation of Innate Lymphoid Cells

**DOI:** 10.3390/ijms20112836

**Published:** 2019-06-11

**Authors:** Grace Mallett, Arian Laurence, Shoba Amarnath

**Affiliations:** 1 Institute of Cellular Medicine, Newcastle University, Newcastle upon Tyne NE2 4HH, UK; g.mallett2@newcastle.ac.uk; 2 Department of Haematology, University College London Hospitals NHS Trust, London NW1 2BU, UK; laurence.arian@icloud.com

**Keywords:** PD-1, ILC, PDL-1

## Abstract

Programmed cell death-1 (PD-1) is a cell surface receptor that dampens adaptive immune responses. PD-1 is activated by the engagement of its ligands PDL-1 or PDL-2. This results in the inhibition of T cell proliferation, differentiation, cytokine secretion, and cytolytic function. Although a great deal is known about PD-1 mediated regulation of CD4^+^ and CD8^+^ T cells, its expression and function in innate lymphoid cells (ILCs) are yet to be fully deciphered. This review summarizes the role of PD-1 in (1) modulating ILC development, (2) ILC function, and (3) PD-1 signaling in ILC. Finally, we explore how PD-1 based immunotherapies may be beneficial in boosting ILC responses in cancer, infections, and other immune-related disorders.

## 1. Introduction

PD-1 is expressed on CD4^+^ and CD8^+^ T cells, B cells, monocytes, natural killer (NK) cells, and dendritic cells (DCs) [1,2,3]. These immune cells have been joined by a novel lineage of innate lymphocytes, key for early responses to infections. PD-1 expression was identified in innate lymphoid cell precursors (ILCp) and our group demonstrated its expression in mature group 2 innate lymphoid cells (ILCs) [4,5,6]. Subsequently, the expression of PD-1 in other ILC subsets has been demonstrated [7]. With the discovery of co-receptors on ILCs, there is a significant possibility that immunotherapies targeting co-receptors may impact ILC function in human disease. Within this review, we explore these opportunities and highlight murine work which has confirmed that ILCs can regulate immune responses through PD-1 in autoimmunity and anti-parasitic diseases. We summarize the current understanding in the field on the role of PD-1 in boosting ILC function in experimental murine models and stress the possibilities of boosting ILC responses by using PD-1-based immunotherapies. We also highlight the lack of understanding within the field on PD-1 signaling in ILCs and the importance of understanding PD-1 biology in order to enhance ILC responses in disease.

## 2. Programmed Death-1 (PD-1)

PD-1 is a member of a family of immunoglobulin domain (Ig) co-receptors that modify the outcome of activation of the T cell receptor by an antigen-presenting cell (APC) or infected target cell. Members of this family can be divided into those that predominantly have a positive effect, driving T cell activation, and those that have a negative effect, restraining T cell activation. The inhibitory role of PD-1 was identified in mice that lacked PD-1 expression that developed autoimmune disease relatively late in life [8]. The expression pattern of PD-1 and its ligands (namely PDL-1 and PDL-2) in various immune cells and tissues suggests that this pathway plays an important role independent of antigen receptors.

### 2.1. PD-1 Function in T Cells

CD8^+^ and CD4^+^ T cell activation through T cell receptor results in the upregulation of the PD-1 receptor in in vitro studies. PD-1 is not expressed on resting T cells but can be induced within 24 h post-stimulation [9]. A similar pattern occurs in APCs [10] and NKT cells [11]. PD-1 binds to two known ligands, PDL-1 (B7-H1; CD274) and PDL-2 (B7-DC; CD273). PDL-1 is constitutively expressed on T and B cells, DCs, macrophages, and in non-hematopoietic cells including keratinocytes, nonparenchymal cells etc. [2] (reviewed in [3]). Emerging data suggest that PD-1 receptor expression contributes towards memory T cell development but an in-depth comparison of T cell function with PD-1 receptor expression kinetics has yet to be fully investigated.

Snapshots of PD-1 receptor expression and its relative contribution towards immune tolerance has been confirmed using animal models of autoimmunity and cancer. However, the molecular mechanisms that control PD-1 expression are relatively unclear. Activated CD4^+^ T cells are known to express inhibitory co-receptors, of which cytotoxic T-lymphocyte associate protein 4 (CTLA4) is the best characterized, as mice that lack this co-receptor die of auto-immune disease within the first few weeks of life [12]. In addition, there is a discrete lineage of CD4^+^ T cells that restrain the immune system; these T regulatory (Treg) cells are characterized by the expression of the master transcription factor Foxp3 and high levels of CTLA4 and CD25. Although PD-1 expression is not a characteristic feature of Treg cells, PD-1 activation has been shown to induce Foxp3 expression and maintain induced Treg cell phenotype [13].

The importance of sustained PD-1 receptor expression in exhausted CD8^+^ T cells was first described in a chronic murine lymphocyte choriomeningitis virus (LCMV) model whereby PD-1 blockade revived CD8^+^ T cell function [14]. Indeed, a comprehensive analysis of PD-1 in chronic viral diseases (including hepatitis B virus (HBV)-specific diseases [15]) has been elucidated [16,17,18,19,20]. These data suggest that T cell receptor stimulation is a driving factor for PD-1 expression in CD8^+^ T cells. Consequently, the idea that PD-1 can be expressed on ILCs that lack antigen-specific receptors was not considered. In addition to T cell receptor activation, other stimuli that induce PD-1 expression include NFATc1 [21], the interferon (IFN) sensitive response element, and signal transducer and activator of transcription 5 (STAT)1/2 in macrophages [22].

### 2.2. PD-1 Structure

PD-1 is a 50–55 kDa type I transmembrane glycoprotein composed of an IgV domain which shares 21–33% sequence identity with other members of the Ig co-receptor family including CTLA4, CD28, and inducible T-cell costimulatory molecule (ICOS) [23]. The structure of PD-1 consists of a single N-terminal IgV domain which is separated from the plasma membrane by a 20-amino-acid stalk, followed by a transmembrane domain and a cytoplasmic tail that contains tyrosine based signaling motifs. Unlike CD28 and CTLA4, PD-1 exists as a monomer as it lacks the membrane proximal cysteine residue required for homodimerization [23]. The PD-1 cytoplasmic tail consists of two tyrosine residues; the membrane proximal one constitutes an immunoreceptor tyrosine-based inhibitory motif (ITIM) and the other an immunoreceptor tyrosine-based switch motif (ITSM), which is required for the inhibitory signals delivered by PD-1 ligation. PD-1 binds to ligands PDL-1 and PDL-2, which results in the recruitment of Src homology region 2 domain-containing phosphatase (SHP)1/2 to the ITSM motif of the cytoplasmic tail. It is yet unclear whether PD-1 signaling preferentially recruits SHP1 or SHP2 to ITSM, with recent reports suggesting a stronger affinity for SHP2 versus SHP1 [24]. These differences make PD-1 an outlier within the Ig co-receptor family, suggesting that PD-1 activation has unique properties unlike CTLA4 (reviewed in [25]). Both PDL-1 [26] and PDL-2 [27] are type I transmembrane glycoproteins composed of IgC and IgV domains and the crystal structure of PD-1 and PDLs has been clarified [28,29]. 

### 2.3. PD-1 Signalling in T Cells

The ITSM is essential for the inhibitory function of PD-1, which was initially demonstrated in B cells [30]. Similarly, in T cells, PD-1 inhibited T cell receptor (TCR) signaling by blocking PI3K, although one study reported that ITSM recruited SHP1 and the other found SHP2 recruitment [9,31,32]. In all of these cases, it was noted that PD-1 activation results in inhibiting TCR response by blocking upstream events post TCR stimulation. A study by Fife et al. demonstrated in addition to biochemical signaling events, PD-1 ligation to PDL-1 inhibited T cell motility and lowered T cell-DC contact [33]. 

The recruitment of SHP1/2 to the ITSM has been analyzed by two groups. In both cases, it was shown that SHP2 had a preferentially binding to the ITSM over SHP1 [9,24] PD-1 activation inhibited both TCR and CD28 signaling in these two studies, suggesting that SHP2 may have a stronger binding to ITSM compared to SHP1 and the relative contribution of each to PD-1 mediated inhibition needs to be further evaluated. In addition to SHP1/2, recent work on identifying PD-1 interactors has identified SAP (SH2D1A) being recruited to the ITSM tail of PD-1 [34]. 

**Figure 1:**
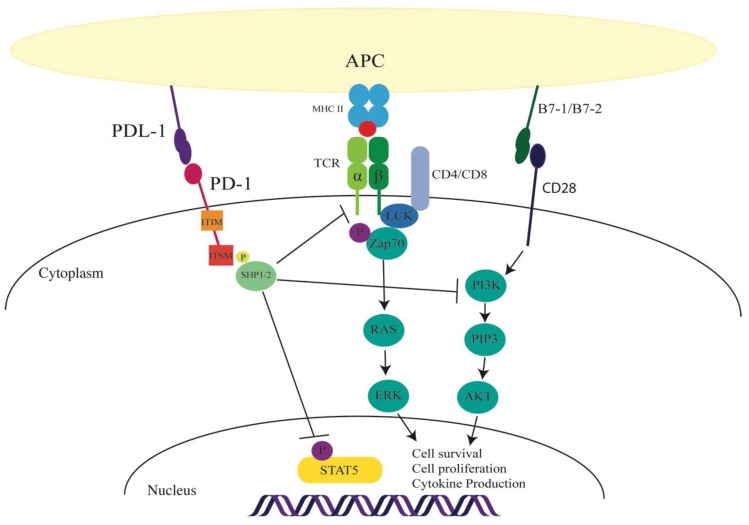
Consequence of programmed cell death-1 (PD-1) activation in immune cells. On engagement of the PD-1 receptor (either through programmed death ligand (PDL) -1 or 2), the PD-1 receptor recruits Sc homology region 2 domain-containing phosphatase (SHP)1/2 to the immunoreceptor tyrosine-based switch motif (ITSM) cytoplasmic tail. PD-1 signaling can inhibit the T cell receptor (TCR) signaling cascade. In hepatitis C virus (HCV) Tregs, the signaling can inhibit signal transducer and activator of transcription 5 (STAT5) phosphorylation. PD-1 inhibition is indicated by T bars; solid arrows indicate TCR and CD28 signaling pathways.

PD-1 signaling in CD4^+^ T cells enhances the induction of Treg phenotype from naïve T cells to form iTreg cells, and participates in human Treg function and in human Th1 to Treg conversion [35,36,37]. Engaging PD-1 downregulated phosphorylation of Akt, mammalian target of rapamycin (mTOR), S6, and extracellular regulated kinase (ERK)2 with a concomitant upregulation of PTEN, suggesting that PD-1 signal promoted iTreg generation by antagonizing the Akt–mTOR signaling pathway. In another study by Franceschini et al. [38], PD-1 inhibited STAT5 signaling, which resulted in Foxp3 destabilization and downregulation of iTreg population, and therefore the first study to identify that PD-1 may regulate STATs in T cells (Figure 1). Indeed, PD-1 activation can inhibit interleukin (IL)-2 production, but the precise signaling mechanism is not clear. In our group, we found that PD-1 can maintain Foxp3 expression in human and murine Th1 cells converted to iTregs [35,36]. These studies implicate dephosphorylation of signaling molecules as a primary mechanism by which PD-1 dampens T cell proliferation and function. The Franceschini study provides the first indication that PDL-1 through PD-1 can regulate similar cytokine signaling pathways within ILCs.

## 3. Innate Lymphoid Cells

ILCs are innate immune cells that play a major role in immune defense and tissue homeostasis. ILCs are classified into three groups: Group 1 ILCs (including NK cells and ILC1s), group 2 ILCs (ILC2s), and group 3 ILCs (including ILC3s and lymphoid tissue inducers (LTi) [39]. Each ILC subset is implicated in specific pathogenic responses mirroring those of cytotoxic T cells and T helper cells. ILCs are concentrated at mucosal surfaces in order to elicit barrier integrity and are defined by three specific features: (1) lack of recombinantion activating gene (RAG)-dependent rearranged receptors, (2) lack of myeloid and dendritic cell lineage-defining factors, and (3) possession of lymphoid morphology. ILCs are activated by cytokines and respond by producing effector cytokines. Both NK cells and LTi cells are well-defined members of ILCs. NK cells were defined in 1975 [40] and mediate important early antigen independent immune responses. In contrast, LTi cells defined in 1997 [41], are vital for formation of lymph nodes during embryogenesis. A summary of the different types of ILCs and their regulation by coinhibitory receptors is discussed below.

### Types of ILCs

ILC classification is based on their cytokine phenotype, function, and master transcription factors that drive their function. A comprehensive summary of ILC subsets, the activating stimuli, effector cytokine production and co-receptor expression is summarized in Table 1. Helper ILC1s are responsible for anti-viral defense and produce type 1 cytokines such as IFNγ [40,42,43]. ILC1s are defined by the expression of the master transcription factor Tbx21 (Tbet) and are activated by cytokines IL-12 and IL-18 [40,42,43], mimicking the Th1 cell phenotype [44,45,46]. ILC2s are defined by the expression of the master transcription factor GATA3 and mirror T helper type 2 (Th2) cells in function. ILC2s are stimulated by alarmins IL-33, thymic stromal lymphopoietin (TSLP), and IL-25 and respond by producing effector cytokines IL-13 and IL-5. ILC2s drive type 2-mediated autoimmune diseases such as allergy and atopic dermatitis. In addition to pathogenic responses, ILC2s can mediate tissue repair through amphiregulin (Areg) and can regulate thermogenesis by converting white fat into beige fat. Group 2 ILCs are further defined as either naïve ILC2s (ST2^+^KLRG1^−^), inflammatory ILC2s (KLRG1^+^ST2^−^), or mature ILC2s (KLRG1^+^ST2^+^) in mice [47,48,49,50,51,52,53]. Group 3 ILCs are sub-divided by the expression of NK receptor NKp46 (in mice) or NKp44 (in humans). Natural cytotoxicity receptor (NCR)^+^ ILC3s are positive for NKp46/NKp44 [44,54,55] whereas NCR^−^ do not express Nkp46/Nkp44 [56]. NCR^−^ ILC3s are associated under normal physiological conditions within the skin, with the upregulation of NCR^+^ cells during inflammatory conditions such as psoriasis [56,57,58]. On the contrary, NCR^+^ ILC3s are the prominent ILC3 population within the intestine of healthy individual. These cells are further defined by the expression of the master transcription factor retinoic-acid-receptor-related orphan nuclear receptor γ (RORγt) and are the innate counterpart of IL-17 expressing T helper (Th17) cells. ILC3s are activated by cytokines such as IL-23 and are efficient at inducing anti-bacterial responses through the production of cytokines such as IL-17 and IL-22. LTi cells are classified within group 3 ILCs, as they share these properties [39]. The classification and sub classification of ILC subsets is an ongoing area of study due to the rising importance of these subsets in diseases, tissue repair and thermogenesis. However, the consensus that ILCs are helper-like immune cells similar to T cells suggests their close resemblance to the adaptive immune arm of the immune system. In recent years, the expression of co-receptors on ILCs have been demonstrated further highlighting the similarities between ILCs and T helper cells.

In addition to their helper like function, emerging literature suggests that ILCs can also mimic APC function through expression of MHC class II which in-turn can modulate T cell function [59,60,61]. Along these lines, recent reports have identified the expression of ligands such as PDL-1 on ILC2s that can modulate T helper function. Within this review a comprehensive analysis of how the PD-1/PDL-1 interactions modulate ILC development and function will be discussed.

## 4. PD-1 and ILCs 

The expression of CD28 co-receptor family on helper ILCs was not considered until recently, when a study by Maazi et al. [62] demonstrated that ILC2s expressed the co-receptor ICOS. ICOS is expressed on activated T cells and is required for the survival and function of T cells, Th2 cell differentiation, and for lung inflammatory responses [63,64,65]. ICOS binds to ligand ICOS-L which results in the secretion of cytokines such as IL-4 and IL-13. ICOS has a similar function in ILC2s and ICOS blockade resulted in downregulating ILC2-mediated lung airway inflammation. At the same time, our group discovered that ILC2 frequency was significantly increased in PD-1^−/−^ mice [6]. This observation was further confirmed by study done by Yu et al. that demonstrated the expression of PD-1 on ILCps. Subsequent reports have identified PD-1 expression on ILC2s, ILC3s, and LTi [7,66]. Furthermore, similar to T cells, PDL-1 expression was also noted in ILC2s by our group and by others [67]. Considering these observations, it can be postulated that ILC2s can be further classified as those that express co-receptors (ICOS, PD-1) or co-receptor ligand (ICOS-L; PDL-1)-expressing APCs like ILC2s. Here, we discuss the current literature describing the relationship between PD-1/PDL-1 signaling and ILC function. We postulate that this may play a significant role in the regulation of these cells. 

### 4.1. PD-1 in ILC Development

The first report that ILCs expressed PD-1 is by Yu et al. [4] where the authors found the expression of PD-1 on ILC precursors using a single cell RNA-seq approach. In this study bone marrow derived ILC precursors were flow sorted based on surface markers (Lin^−^Flt3^lo/−^IL7Rα ^lo/+^α4β7^+^) and then subjected to single cell RNA-seq. The authors found that within the common helper innate lymphoid progenitors (CHILP) identified as Lin^−^CD25^−^IL7Rα^+^α4β7^+^ and classified as cluster 6, also expressed PD-1. This PD-1^hi^ population conformed to a progenitor phenotype concurrently expressing *Zbtb16, Id2, Tcf7, Tox,* and *Gata3* (reviewed in [68]) and lacked mature ILC2 markers in addition to the absence of *pdcdlg1* or *pdcdlg2*. These progenitors could differentiate into various ILC subsets on adoptive transfer into a *Rag*^−*/*−^*γc*^−*/*−^ mice but did not generate T or B cells. However, a similar study done by Seillet et al. [5] demonstrated that the transfer of PD-1-deficient ILC progenitors were similarly capable of generating ILC subsets on adoptive transfer; similarly, no difference in ILC development was noted within our own study. Indeed, the Yu et al. study confirms that PD-1 does not have a functional role in ILC development but is an activation marker and can be efficiently utilized for identifying and isolating CHILPs or ILC2 precursors that are *bcl11b*^+^ and *Il17rb*^+^. Deleting *bcl11b* completely inhibited ILC2 development from the PD-1^hi^ compartment, however overexpressing *Il17rb* within this compartment restored ILC2 development. The question remains as to the stimuli that induces PD-1 on ILC precursors and whether PD-1 expression is driven by *γc* cytokines. 

### 4.2. PD-1 Modulation of ILC Function

**Figure 2:**
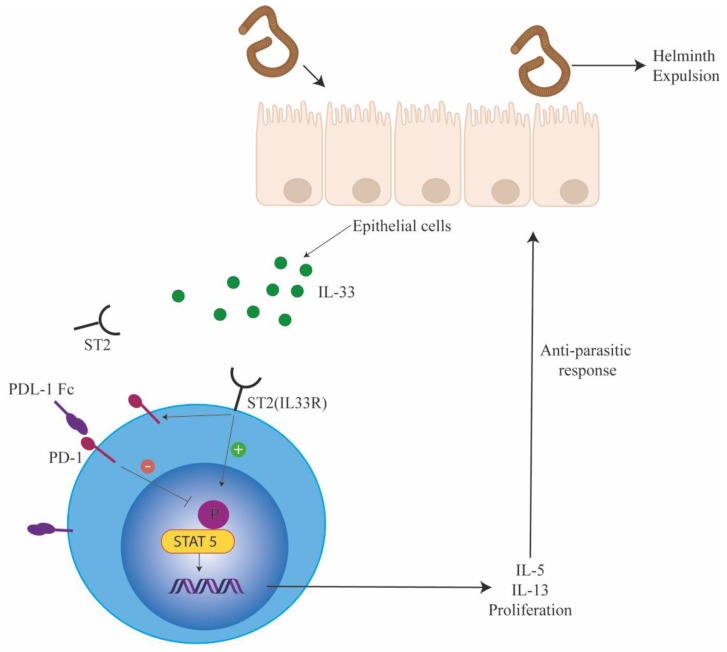
Innate lymphoid cells (ILC2s) are negatively regulated by PD-1. ILC2s are important for eliciting defense against parasitic infection. During parasitic infections, alarmins such as IL-33 are released by the gut epithelia cells. IL-33 activates ILC2s by binding to the IL-33R. On activation, ILC2s secrete type 2 cytokines that mediate Th2 responses, resulting in helminth expulsion. In addition, IL-33 also induces PD-1 receptor on ILC2s as a regulatory feedback loop (solid arrows). PD-1 dampens ILC2 proliferation and function on binding to its ligand PDL1 (inhibition shown by T bar).

PD-1 expression on mature ILCs was also reported by Yu et al. [4], whereby PD-1 expression was distributed between ILC2s (20–40%), ILC3s (20–30%), and small intestine lamina propria LTi cells (76%) but not in conventional natural killer (cNK) or ILC1 cells. A substantial increase in PD-1 expressing ILC2s were noted on challenge with influenza infection and this population was also known to express IL-13. Similar to this work, our group demonstrated that PD-1 regulated ILC2 function during parasitic helminth infections (Figure 2). We found that PD-1 expression was significantly driven by IL-33 and absence of PD-1 increased ILC2 proliferation and function. To further clarify the role of PD-1 in ILC2 function, we tested the efficacy of PD-1 blockade in eradicating helminth worms in *Rag*^−*/*−^ mice. We found that blocking PD-1 significantly enhanced the expulsion of worms in *Rag*^−*/*−^ mice. However, this experimental set up included blocking PD-1 in both myeloid and ILC compartments. The function of PD-1 on ILC2 was confirmed as follows: *Rag*^−*/*−^*γc*^−*/*−^ mice infected with *Nippostrongulus brassiliensis* were reconstituted with either wildtype(WT) or PD-1^−/−^ ILC2s. Within this experimental condition, we found that PD-1 deficient ILC2s were significantly superior to WT ILC2s in diminishing worm burden. Blocking PD-1 also enhanced human ILC2 function both in vitro and in vivo suggesting a conserved PD-1 mediated regulatory function in ILC2s. Traditionally associated as a T cell targeting therapy, we describe here a potential novel use of PD-1 blockade to target ILC2s in the context of helminth infection; which was also eluded to by Yu et al. in their model of influenza. Our study also confirmed murine findings in human system where PD-1 blockade enhanced ILC2 function. These combined studies open up a new are of immunotherapy for parasitic helminth disease whereby checkpoint blockade can enhance ILC2-mediated immune responses to parasites. Indeed, one needs to be cautious with such therapies due to their deleterious effects in inducing airway inflammation.

Recently, Oldenhove et al. [66] demonstrated that PD-1 expression on ILC2s can result in the dysregulation of tissue metabolism. ILC2s are vital for the conversion of white fat into beige fat thereby limiting adiposity. PD-1 engagement of ILC2s to PDL-1 on M1 macrophages rendered ILC2 dysfunctional in mice fed with a high-fat diet. These observations highlight a possible role for PD-1 in adipose tissue metabolism whereby blocking PD-1 can enhance ILC2 function resulting in the conversion of mitochondrial poor white fat to mitochondrial rich brown fat. Of note, the work by Oldenhove confirmed our findings that IL-33 plus IL-2 and IL-7 were capable of inducing PD-1 on ILC2s. The work further extended this observation by demonstrating that the cytokine tumor necrosis factor (TNF), through IL-33, induced PD-1 expression on ILC2s.

The expression of PD-1 on ILC3 and LTi has been recently reported in the human decidua. In this study, the authors sequentially measured PD-1 expression in the maternal ILC compartment during the first and the third trimester. During the first trimester PD-1 was highly expressed on LTi while expression was also noted on ILC3s. In the third trimester, PD-1 expression was significantly downregulated in the LTi cells but this expression was similar to that noticed in ILC3s. Although NK cells lacked PD-1 expression in the first trimester, they were able to significantly upregulate PD-1 in the third trimester. However, PD-1 expression on NK cells did not reach the same frequency as LTi, ILC3, or T cells. The expression of PDL-1 was restricted to the intermediate extravillous trophoblast (iEVT) at the intersection of the feto-maternal interface, suggesting an ILC mediated tolerance mechanism driven by PD-1/PDL-1 in order to prevent fetal rejection in the early phase of pregnancy [7]. Further work is required in order to isolate the functional relevance of PD-1 expression in ILC3s and particularly the consequence of PD-1 expression kinetics on ILCs throughout pregnancy. 

### 4.3. PD-1 Signalling in ILCs

Emerging evidence of PD-1 expression on ILCs indicate that PD-1 signaling can inhibit ILC function. The precise molecular mechanisms that induce PD-1 on ILCs or the signaling pathways that are activated by PD-1 engagement is yet to be fully understood. While our study and the work of others have shown that PD-1 can be induced by ILC2 and γc cytokines, the precise molecular events that induce PD-1 has not been delineated in ILC2s. 

In order to elucidate the molecular signaling pathways that are modulated by PD-1 engagement, we explored the T cell literature to identify relevant PD-1 modulating signaling pathways that might be operational in ILC2s. The work by Franceschini et al. [38] on HCV suggested that PD-1 signaling can inhibit STAT5 phosphorylation in Tregs. Hence, we explored if PD-1 engagement downregulated STAT5 phosphorylation in ILC2s. For these experiments, ILC2s were isolated from WT and PD-1 deficient mice and then stimulated with IL-2 ex vivo followed by measurement of pSTAT5. A significant increase in STAT5 phosphorylation was noted PD-1 deficient ILC2s as compared with WT ILC2s. In addition, we found in our in vitro cultures, engaging PD-1 with plate coated PDL-1 fc resulted in the downregulation of pSTAT5 both in mice and human ILC2s [6]. These experiments suggest that PD-1 engagement downregulates STAT5 phosphorylation in ILC2s and consequently inhibits ILC2 proliferation and function. However, a comprehensive proteome and phospho-proteome analysis is required to fully understand the molecular signaling pathways targeted by PD-1 engagement in ILC2s. 

### 4.4. PD-1 Modulation of ILC in Cancer

In light of the success of PD-1 blocking antibodies in cancer immunotherapy, it remains possible that in addition to enhanced T cell responses, PD-1 may regulate the helper ILC arm of the immune system to modulate anti-tumor responses. The function of helper ILCs in anti-tumor responses has been recently explored whereby ILC1s possess both pro [43] and anti-tumor function [69]. In addition, a recent study on human breast cancer patients have shown a significant increase in ILC2s in malignant tissue as compared to benign tumor tissue. However, PD-1 expression was uniformly increased in ILC2 within both benign and malignant breast tissue. In contrast, within gastrointestinal tumors, a significant increase in PD-1 expression was noted in ILC2, ILC3, and NK cells [70]. PD-1 expression was also demonstrated in NK cells and ILC3s within the malignant pleural effusion taken from both primary and metastatic tumors [71]. This study simply correlated PD-1 expression in tumor tissue, but a significant understanding PD-1 signaling within tumor ILCs is required prior to determining the efficacy of PD-1 based therapies in modulating ILCs in tumor tissue. A systematic analysis of ILC function in tumor followed by the regulation of PD-1 of tumor derived ILCs is warranted. However, it is beyond doubt that the work by Salimi et al. has highlighted a role for PD-1 in regulating ILCs within cancers. Indeed, ILC3 can sustain colon carcinoma through the production of IL-22 [72]. In this instance PD-1 expression within ILC3s may dampen the inflammatory microenvironment by inhibiting the function of ILC3s. It is hence important to determine the precise function of PD-1 on ILCs within the tissue specific tumor microenvironment. Furthermore, ILCs may occupy the niche within non-immunogeneic cancers that do not manifest significant T cell infiltration. ILCs are tissue resident immune cells and their function can be boosted by PD-1 therapies in tumors that are poorly immunogeneic thereby adding a relatively new functional immune arm within cancer immunotherapy.

## 5. Conclusions

In summary, ILCs have emerged as an important component of the innate immune system that can influence adaptive immune responses, regulate tissue repair and adipose tissue. The expression of PD-1 on ILCs has identified a new therapeutic window in non-immunogeneic and immunogeneic tumors that needs to be further elucidated. These studies highlight the finding that the role of these receptors is broader than simply modifying the action of antigen receptors. A full comprehensive understanding of ILC modulation by PD-1 will be required in order to fully utilize this signaling pathway in the clinic. Given the number of clinical trials investigating anti-PD-1, PDL-1, and CTLA4 antibodies, it is essential to understand how these interventions affect ILC function within the tumor tissue. 

## 6. Future Perspectives

With the discovery of ILCs, we have the potential for boosting or dampening tissue-specific immune responses in the context of diseases such as cancer, autoimmunity, and infectious pathogens. Finetuning these responses requires an in-depth understanding of ILC immunobiology and the impact of immune-therapeutics such as jakinib and checkpoint inhibitors in boosting specific ILC responses. Since immunotherapeutic platforms are already well established in cancer and autoimmune diseases such as rheumatoid arthritis, it will be possible to tease out ILC responses to these various therapies in the clinic. Therefore, it is important to include ILCs as a target immune population within these clinical trials in order to fully understand the contribution of these subsets in human disease. In addition, the use of ILCs as a cellular therapy in diseases such as graft-versus-host disease has been recently proposed. The efficacy and clinical feasibility of this process has yet to be fully established [73]. Indeed, prior to using ILCs as cellular therapeutics, it would be important to decipher ILC immunobiology and fully understand the regulatory networks that dictate ILC proliferation and function in vivo. 

## Figures and Tables

**Table 1:** Summary of Innate Lymphoid Cell subsets and function.

Group	Subsets	Transcription Factor Expression	Co-receptor Expression	Cytokine/Alarmins Stimulation	Effector Cytokine Profile	Role Within the Immune System
Group 1 ILC	NK Cells (CD49b^+^) ILC1(Lin^−^CD127^+/-^CD49a^+^)	Tbet^+^EOMES^+^ Tbet^+^	PD-1 CTLA4	IL-15 IL-12 IL-18	IFNγ TNFα Perforin and granzyme (NK cells)	Viral defence Anti-Tumour defence Intestinal inflammation
Group 2 ILC	Natural (ST2^+^, IL33R^+^) Inflammatory (KLRG1^+^) Mature (ST2^+^KLRG1^+^)	GATA3^+^	PD-1 CTLA4 ICOS (PDL-1) (OX40L)	IL-25 IL-33 TSLP	IL-4 IL-5 IL-13 Amphregulin	Helminth defence Thermogenesis Tissue repair Allergy Atopic dermatitis
Group 3 ILC	NCR^+^ (Nkp46^+^/Nkp44^+^) NCR^−^ (Nkp46^−^/Nkp44^−^) LTi	RORγt^+^	PD-1 CTLA4	IL-23 IL-1β	IL-17 IL-22 GM-CSF	Intestinal homeostasis Bacterial defence Colitis Psorasis Colon Cancer
